# Contribution of federal health expenditure accounts to prevention reporting: possible approaches

**DOI:** 10.17886/RKI-GBE-2017-094

**Published:** 2017-08-30

**Authors:** Susanne Meise, Miriam Orlowski

**Affiliations:** 1 Federal statistical office of Saxony, Department 34 – Economic analyses, national accounts, Germany; 2 Federal statistical office of Bavaria, Department 32 – National accounts, employee statistics, Germany

## Abstract

The health economic national accounts of federal states working group provides data on the annual health expenditure of federal states, health care sector employee figures as well as on gross value added and health economy personnel. Based on these results, federal health reporting can provide data for various indicators (health sector personnel, expenditure and funding). Today health expenditure accounts data and/or additional data sources provide a basis to determine expenditure on disease prevention and health protection for each federal state. These results are highly relevant to the field of prevention reporting.

## Understanding health expenditure accounts

The health economic national accounts of federal states working group (Arbeitsgruppe Gesundheitsökonomische Gesamtrechnungen der Länder, AG GGRdL), which was founded in 2009 and is headed by the federal statistical office of Saxony, is responsible for providing health sector data that covers all federal states. This data is collected as part of health expenditure and health personnel accounts in accordance with the System of Health Accounts (SHA). Currently, the working group contributes three figures: health expenditure accounts calculated for budget holders, health personnel accounts calculated for each institution and figures on gross added value and health economy personnel. Given the working groups remit of providing data for federal state level expenditure on disease prevention, this article focuses on health expenditure accounts.

Health expenditure is calculated based on the SHA concept, which is recommended by the Organization for Economic Co-operation and Development (OECD) to ensure the international comparability of data [1]. It includes all expenditure on goods and services in the context of disease prevention, treatment, rehabilitation and care, as well as investments in health care institutions [2]. SHA identifies three dimensions – health expenditure calculated for budget holders, as well as for each service and institution type – and therefore aims to answer the following question: What is funded by whom and where is the service provided? Currently, at the federal level, health expenditure data is only collected on a differentiated basis for budget holders.

## Federal state level health accounts expenditure and prevention reporting

Based on federal level calculation methods and carried out in line with SHA specifications, the AG GGRdL approach covers all federal states, ensuring the full comparability of data provided for federal states and at the national level. Calculations are based on a broad set of data sources. This includes data from official statistics such as the statistics on expenses for asylum seekers and social benefit statistics, account data from the National Association of Statutory Health Insurance Funds (GKV-Spitzenverband) as well as data from the Federal Employment Agency. The methodology and data sources are documented transparently and published on the working group’s website [3].

Table 1 shows the 2014 budget holder health expenditure calculations for the eleven AG GGRdL member states and Germany. Statutory health insurance funds are the largest budget holder and accounted for nearly 60% of total health expenditure in Germany in 2014. This fact particularly emphasises the need for a regionally differentiated approach. Whereas in Saxony over 65% of total expenditure is borne by statutory health insurance funds, this share is 10% lower in Bavaria.

The planned further development of health expenditure accounts at the federal level foresees calculating expenditure separately according to the type of service. In accordance with SHA, services can be classed, for example, as disease prevention and health protection services. Until health expenditure accounts calculated for specific services have been fully established, an evaluation of GKV-Spitzenverband account data regarding expenditure on disease prevention and health protection could potentially be rapidly made available for both budget holders (statutory health insurance and nursing care insurance). Analogous to federal level accounts, these accounts also respect SHA guidelines.

## Outlook

In future, it will be possible to provide accounts on disease prevention and health protection that are divided according to federal state for all budget holders. Federal state level health expenditure accounting will then provide an important contribution for prevention reporting.

## Figures and Tables

**Table 1:** Health expenditure in Germany and select federal states by budget holder (2014) Data sources: federal state health expenditure accounts [3], national health expenditure accounts [4], last updated March 2016

Federal state Federal government	Total	as a percentage of			
		Public budget	Statutory health insurance funds	Private health insurance funds	Further budget holders
	In million €	Share of total in %			
Baden-Württemberg	41,851	4.0	55.9	10.5	29.6
Bavaria	50,896	4.6	55.3	10.6	29.5
Berlin	14,144	5.8	58.8	8.9	26.5
Brandenburg	10,387	3.4	64.0	7.2	25.4
Hamburg	6,812	6.4	55.9	10.8	26.9
Hesse	24,522	4.6	57.0	9.9	28.5
North Rhine-Westphalia	71,009	4.8	58.4	9.0	27.8
Rhineland Palatinate	16,335	4.4	55.7	10.3	29.6
Saxony	16,597	3.4	65.6	4.7	26.3
Schleswig-Holstein	11,206	4.7	57.6	9.7	28.0
Thuringia	8,707	3.8	65.4	5.3	25.5
**Germany**	**327,951**	**4.5**	**58.5**	**8.9**	**28.1**
